# LupiQuant: A real-time PCR based assay for determining host-to-parasite DNA ratios of *Onchocerca lupi* and host *Canis lupus* from onchocercosis samples

**DOI:** 10.1371/journal.pone.0276916

**Published:** 2022-11-21

**Authors:** Chandler C. Roe, Jennifer Urbanz, Candace Auten, Guilherme G. Verocai, Kelly Upshaw-Bia, Olivia Holiday, Crystal Hepp, Jason W. Sahl

**Affiliations:** 1 The Pathogen and Microbiome Institute, Northern Arizona University, Flagstaff, AZ, United States of America; 2 School of Informatics, Computing, and Cyber Systems, Northern Arizona University, Flagstaff, AZ, United States of America; 3 Eye Care for Animals, Scottsdale, AZ, United States of America; 4 Eye Care for Animals, Albuquerque, NM, United States of America; 5 Department of Veterinary Pathobiology, College of Veterinary Medicine & Biomedical Sciences, Texas A&M University, College Station, TX, United States of America; 6 Navajo Nation Veterinary Management Program, Window Rock, NM, United States of America; 7 Pathogen and Microbiome Division, Translational Genomics Research Institute, Flagstaff, AZ, United States of America; Visayas State University, PHILIPPINES

## Abstract

*Onchocerca lupi* is a filarial nematode that causes ocular onchocercosis in canines globally including North America and areas of Europe, North Africa, and the Middle East. Reported incidence of this parasite in canines has continued to steadily escalate since the early 21^st^ century and was more recently documented in humans. Whole genome sequencing (WGS) of this parasite can provide insight into gene content, provide novel surveillance targets, and elucidate the origin and range expansion. However, past attempts of whole genome sequencing of other *Onchocerca* species reported a substantial portion of their data unusable due to the variable over-abundance of host DNA in samples. Here, we have developed a method to determine the host-to-parasite DNA ratio using a quantitative PCR (qPCR) approach that relies on two standard plasmids each of which contains a single copy gene specific to the parasite genus *Onchocerca* (major body wall myosin gene, myosin) or a single copy gene specific to the canine host (polycystin-1 precursor, *pkd1*). These plasmid standards were used to determine the copy number of the *myosin* and *pkd1* genes within a sample to calculate the ratio of parasite and host DNA. Furthermore, whole genome sequence (WGS) data for three *O*. *lupi* isolates were consistent with our host-to-parasite DNA ratio results. Our study demonstrates, despite unified DNA extraction methods, variable quantities of host DNA within any one sample which will likely affect downstream WGS applications. Our quantification assay of host-to-parasite genome copy number provides a robust and accurate method of assessing canine host DNA load in an *O*. *lupi* specimen that will allow informed sample selection for WGS. This study has also provided the first whole genome draft sequence for this species. This approach is also useful for future focused WGS studies of other parasites.

## Introduction

*Onchocerca lupi* is a filarial nematode that represents an emerging threat to wildlife, companion animals, and humans [1]. First described in the Republic of Georgia in the periocular tissues of a wolf (*Canis lupus lupus)* in 1967 [2], it was only recently detected in domesticated canines and felines in North America and the Old World [3–5]. As there is no current commercial diagnostic test for this parasite, *O*. *lupi* infections are confirmed based on ocular nodules on eyelids, conjunctiva, and sclera [6–8]. If nodules are not present but *O*. *lupi* infection is suspected, the only diagnostic tool currently available is through the detection of microfilariae in skin [9]. However, this invasive skin biopsy is heavily dependent on the biopsy location and the density of microfilaria [10] making this tool highly unreliable.

*Onchocerca lupi* poses a new public health and veterinary threat, but the genomic mechanisms that drive the evolution of pathogenicity are largely unexplored. Because of the growing number of both canine and human cases of *O*. *lupi*, it is imperative to understand the genomic content of this parasite to identify appropriate and *O*. *lupi* specific biomarker targets, mitigation strategies, and effective treatments. To date, there are no evidence-based treatment protocols for adult *O*. *lupi* nematode infections. Current treatment methods are based mostly on *O*. *volvulus* and involve the anti-microfilaricidal drug ivermectin concurrently given with the antibiotic doxycycline [11, 12]. However, there is no cure for this filarial nematode, highlighting the dire need for novel treatment therapies for this emerging parasite; one way to achieve this is by characterizing the whole genome of the parasite itself. Previous research has shown the production of draft genomes for filarial nematodes has significantly contributed to the identification of potential new drug treatment options [13, 14]. To date, there are no studies investigating the genomic landscape of *O*. *lupi* beyond mitochondrial genes. However, a recent study involving the closely related *Onchocerca ochengi*, a cattle parasite, described the sequencing of 20 whole genome samples and subsequently reported that data from 10 of those samples were majority host (cattle) DNA and therefore unusable [15]. To circumvent costly sequencing of majority host DNA in *O*. *lupi* samples, we have designed a qPCR to quantify the ratio of *O*. *lupi* parasite DNA and *Canis lupus familiaris* host DNA within a parasite sample. Here, we implemented a previously published [16] approach based on single copy genes unique to the parasite (*myosin*) or the host *(*polycystin-1 precursor, *pkd1)* [17]. Additionally, the highly conserved *pkd1* gene target can be used to quantify DNA ratios from coyote, wolf, and dingo samples in addition to canine hosts. This approach is crucial for informed sample selection for whole genome sequencing of parasitic nematode samples that will allow for the development of novel, species-specific biomarkers for pathogen tracking, identify potential treatment targets, and determine population structure and evolution of this newly emerging zoonotic parasite. Additionally, this study produced the first draft genome for this species using this approach.

## Materials and methods

### Single copy gene target selection

The polycystin-1 precursor (*pkd1*) canine gene (accession no. AF483210) was identified as a conserved, single copy gene in a previous study [17] and was selected for use as the host locus based on these criteria. Pre-aligned *pkd1* canine gene sequences (n = 4) (S1 Table) were downloaded from the NCBI nucleotide database [18] and used for primer design in the online software Primer3 [19]. The parasite locus, major body wall myosin gene, was chosen as it was a highly conserved, single copy gene across the genus *Onchocerca*. Briefly, the genomic sequences for 4 *Onchocerca* species (*O*. *ochengi*, *O*. *flexuosa*, *O*. *volvulus*, and *O*. *lupi*) (S2 Table) were aligned to predicted coding sequences pulled from *O*. *ochengi* (accession no. ASM90053720v1) reference genome using BWA v0.7.17-r1188 [20] within the NASP v1.2.0 pipeline [21]. Nucmer v3.1 [22] was used to identify single copy coding regions within the reference genome. Coding regions that were highly conserved across all *Onchocerca* genomes were considered for primer design using Primer3 software.

### Sample collection and DNA isolation

Single copy host and parasite genes were amplified from a pre-established *O*. *lupi* PCR-positive canine skin biopsy sample collected from northern Arizona, United States under IACUC of Northern Arizona University approved protocol 19–016. Genomic DNA was extracted from a complex, biopsied canine skin sample using the Qiagen Blood and Tissue Kit (Qiagen) following overnight lysis, according to the manufacturer’s recommendations. *O*. *lupi* DNA was confirmed using previously published methods [1]. Additionally, four adult *O*. *lupi* isolates from four dogs in Flagstaff, Arizona; Phoenix, Arizona; and Albuquerque, New Mexico (n = 2) were used in this study. Adult worms contained within host tissue nodules were isolated using a 0.3% collagenase enzymatic digestion to remove host tissue and subsequently washed four times with PBS [11]. Genomic DNA was extracted using a modified filarial parasite genomic DNA isolation protocol [23] as follows: samples underwent three freeze/thaw cycles consisting of three minutes in liquid nitrogen followed by three minutes at 80°C. Afterward, samples were transferred to 2mL round bottom tubes with a single 5mm stainless steel bead, 250μL PBS, and 100μL lysis buffer. Using a vortex mixer with a special adapter, samples were vortexed on max speed for 45 min with rotation of the tubes every 10 minutes. Immediately following bead beating, 30μL of 10% SDS was added to each sample along with 2μL of 2−mercaptoethanol and 60μL of proteinase K (20mg/μL). Samples were incubated overnight at 65°C followed by an RNase A treatment which consisted of adding 15μl of RNase A (10mg/mL) to each sample and incubated at 37°C for one hour. The Qiagen DNeasy Blood and Tissue kit (Qiagen) was used following the manufacturer’s recommendations with one exception; buffer AL was added at a 1:1 ratio with the sample volume. Extracted DNA was stored at -20°C until further use.

### Cloning of the *pkd1* and the myosin gene

The *pkd1* and myosin plasmid construct genes were amplified from the *O*. *lupi* PCR-positive canine skin biopsy sample using the thermocycler conditions below:

*pkd1*: 95°C for 3 min, followed by 40 cycles of 95°C for 30 seconds, 60°C for 30 sec, 72°C for 1:30 min and a final extension of 72°C for 1 min.

myosin: 95°C for 3 min, followed by 35 cycles of 95°C for 30 seconds, 60°C for 30 sec, 72°C for 1:30 min and a final extension of 72°C for 1 min.

gDNA from a PCR-positive *O*. *lupi* canine skin sample was used as a template for both reactions and no template controls were included for all PCRs. Primer sequences for both the plasmid construct amplicons as well as the SYBR assay targets are given in Table 1. Non-specific banding was observed in the plasmid construct *pkd1* gene PCR; therefore, the PCR product just below the 1000base marker was extracted from a 2% agarose gel and purified using the QIAquick Gel Extraction Kit (Qiagen). Both the *pkd1* and myosin amplified product were ligated into a TOPO TA vector (Invitrogen) according to manufacturer’s instructions. Plasmid constructs were amplified through transformation into One Shot TOP 10 chemically competent *E*. *coli* (Invitrogen) followed by overnight culturing. To ensure plasmid stability, ten individual colonies were re-streaked on LB containing 50mg/mL kanamycin and incubated overnight at 37°C. Cells were harvested and the cloned plasmid was extracted using the QIAquick Mini Kit (Qiagen). Gene inserts were confirmed for each plasmid by restriction digest using ECORI and sequenced directly with capillary electrophoresis using BigDye Terminator v3.1 Cycle Sequencing Kit on a 3130 Genetic Analyzer platform (Applied Biosystems) using M13 Forward and M13 Reverse (M13 FR) vector primer sites for all replicates. All sequences were queried with blastn [24] against the NCBI Nucleotide database (nt) to confirm the composition of gene targets within each plasmid.

**Table 1:** Primer sequences for quantitative and conventional PCR for both host and parasite used in this study.

Target	Goal	Sequence 5’-3’	Product Size (bp)
*pkd1*	plasmid	F: GGCCATAGTCAATTCCAGCG	951
		R: CCCAGATCATTGAAGGCACG	
*pkd1*	qPCR	F: ACATAGACCGCGGCTTCG	336
		R: TGACCTGCAGATGGAAGCG	
myosin	plasmid	F:GGATATCGCTGGATTCGAGA	991
		R:CGGTCATGCTATCATGGAAA	
myosin	qPCR	F:AACGCGAAGGTATTCAGTGG	339
		R:GATCATTCGCTTTAGATTGTTTCA	

### qPCR SYBR green based assay

Internal primers for use with SYBR dye-based qPCR assays were designed using Primer3 software for both *pkd1* and myosin genes. All qPCR assays were performed on an Applied Biosystems QuantStudio 12. Concentrations for the plasmid preps were measured using the Qubit dsDNA BR assay kit (Invitrogen). A fresh tenfold serial dilution ranging over six logs (10^6^ to 10 gene copy number (GCN)) of both the pTOPO-*pkd1* and pTOPO-myosin plasmids were used to generate each standard curve (Table 1). A 10μL qPCR mixture was prepared using the PowerUp SYBR Green Master Mix (Invitrogen): 1X PowerUp SYBR Green Master Mix, 0.3μM forward and reverse primers, and 2μL template DNA or plasmid standards. The thermal cycling protocol was as follows:

95°C for 10 min, followed by 40 cycles of 95°C for 15 sec, 60°C for 1 min.

Following amplification, a melting curve analysis was used to confirm reaction specificity; a single and specific peak was generated for each primer pair. Both negative and no-template controls were performed in triplicate.

#### Estimation of gene copy number

The gene copy number (GCN) of both plasmids used in this study were calculated using the following equation [25]:

$$GCN=\frac{6.02\;X\;10^{23}\frac{copy}{mol}\;X\;DNA\;amount\;(g)}{DNA\;length\;(bp)X\;660(\frac{g}{mol/bp})}$$

DNA length represents the combined length of the plasmid (3,931 bp) and corresponding insert (*pkd1* = 951 bp, *myosin* = 991 bp). The DNA amount represents the plasmid concentration multiplied by the volume used. Standard curves were generated using copy number vs. C_q_ value for all six plasmid dilutions in triplicate for both pTOPO-*pkd1* and pTOPO-*myosin* plasmids.

#### Host and parasite DNA ratio calculations

GCNs estimated from the pTOPO-*pkd1* and pTOPO-*myosin* standard curves were used to calculate total host and parasite DNA in four *O*. *lupi* samples. Given the estimated genome sizes of host (2370Mb) and parasite (150Mb) as well as the estimated gene copy numbers, we used the above equation to solve for the “DNA amount” in grams. The following equation was used to calculate host to parasite DNA ratio:

Ratio=[ParasiteDNA/TotalDNA(Host+Parasite)]x100

### DNA sequencing and analysis

Four *O*. *lupi* DNA samples extracted from adult nematodes were prepared for paired-end, whole genome sequencing on either a MiSeq, HiSeq, or NextSeq using previously described methods [26]. To aide in the creation of a reference genome, sample Olupi_Ro2020_NM was sequenced on both Illumina NextSeq and MiSeq instruments. Raw reads were trimmed for adapter sequences using trimmomatic v0.39 [27]. The first draft genome assembly for this species was created using SPAdes v3.15.3 [28]. Assembly errors were corrected with eight rounds of pilon [29]. Reads were globally aligned to both the dog reference genome (accession number GCA_008641055.1) and our *O*. *lupi* draft assembly (BioProject PRJNA802584) using bowtie2 v.2.4.2 [30] default parameters with the addition of -I 125, and -X 1800. The number of aligned reads to each reference genome were calculated using PICARD tools v1.125 [31].

## Results

### Melting temperature and standard curve analysis

Two plasmid standards each containing a highly conserved gene specific to the canine host (951 bp fragment of the *pkd1* gene) or the parasite (991 bp fragment of the *myosin* gene) were constructed. The amplicon targets used for the qPCR assay are nested within larger gene fragments (Table 1). These nested primers produced a single amplicon for each gene target, *pkd1* (336bp) and *myosin* (339bp) (S1 Table). Melting curve analysis for both *pkd1* and myosin amplicons revealed single peak temperature at 92°C and 81°C respectively for the standard plasmid DNA and the 3 biological samples.

Standard curve slopes were -3.499 for *pkd1* plasmid and -3.509 for the myosin plasmid. Regression analysis was used to evaluate prediction accuracy which resulted in R^2^ values of 0.999 (*pkd1*) and 1.0 (*myosin*) and standard curve efficiencies of 93.11% and 92.74%, respectively. No template controls were included in each run to ensure PCRs were contamination-free.

### Host-to-parasite DNA ratio predictions

**Fig 1:**
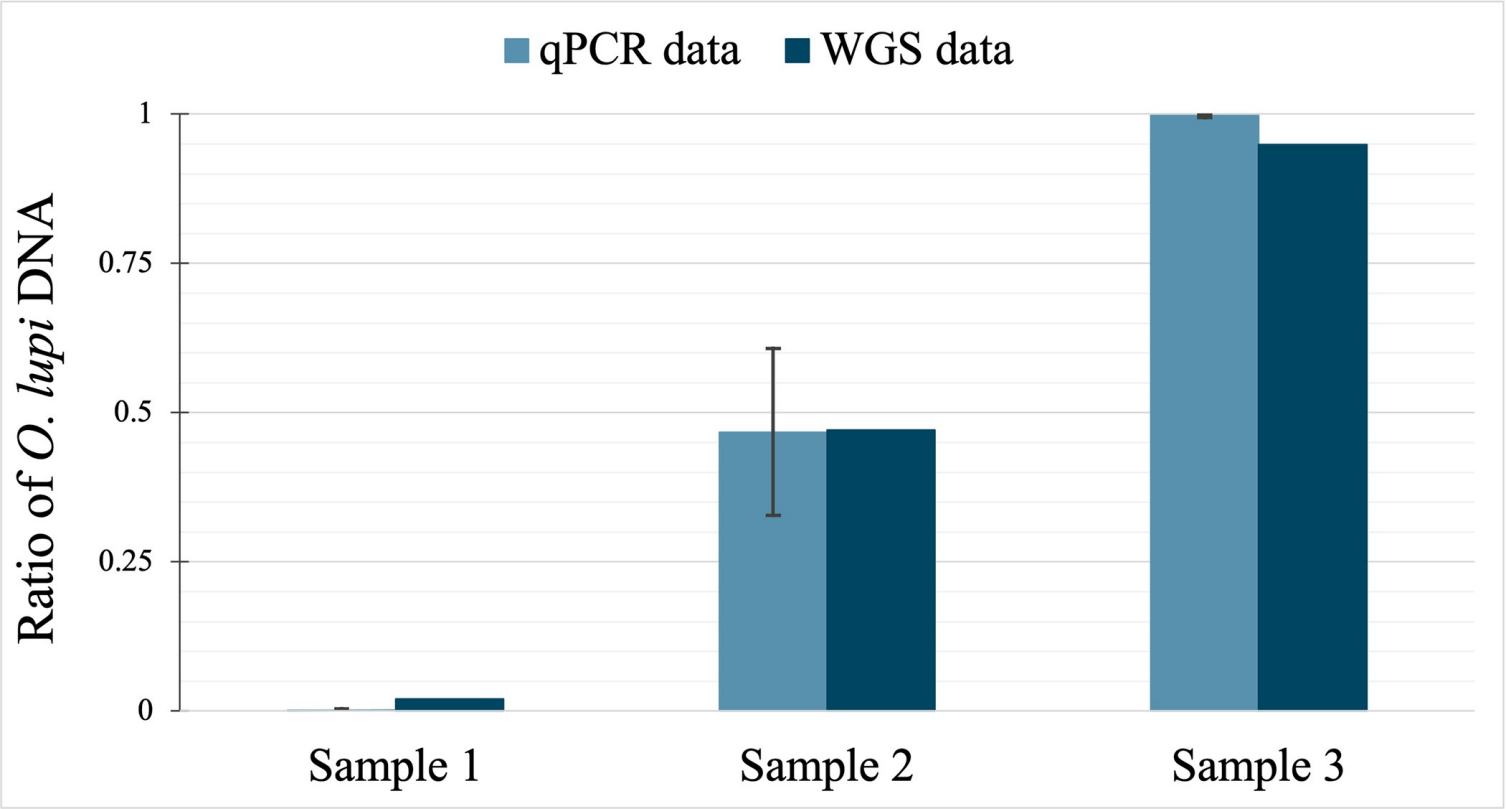
Comparison of *Onchocerca lupi* percentage from three biological canine samples. Samples were screened using LupiQuant which estimated *O*. *lupi* percentages within each sample. Whole genome sequences of *O*. *lupi* samples were aligned to *O*. *lupi* reference genome (BioProject PRJNA802584) to compare LupiQuant ratios. Error bars represent the range of *O*. *lupi* percentages based on host and parasite technical replicates.

Using DNA extracted from three *O*. *lupi* isolates, host and parasite DNA ratios were estimated using LupiQuant. Standard curves were plotted using the *pkd1* and *myosin* plasmid constructs; GCN of the host and parasite DNA per sample were calculated using the C_q_ values in reference to the standard curves. The host to parasite DNA ratio was calculated using equation 2. In the three *O*. *lupi* samples used for quantification, parasite DNA (%) ranged from 0.12% to 46.75% to 99.74% (Fig 1).

#### Whole genome sequencing and DNA ratios

Aligned read counts were tallied and aligned read percentages were compared with the predicted host-to-parasite DNA ratios (Table 2). The WGS data (BioProject PRJNA802584) for three samples showed the parasite DNA alignment (% of total reads) ranged between 1.98% to 47.15% to 94.45% (Fig 1).

**Table 2:** qPCR predicted host-to-parasite DNA ratios, WGS read alignment percentages, and from three adult *O*. *lupi* nematodes. *Reported ratio is not based on total read numbers.

Sample	Predicted DNA ratio (host to parasite)	WGS alignment ratio (host to parasite)
Isolate 1	99.88: 0.12	99.48: 1.98
Isolate 2	53.25: 46.75	52.85: 47.15 *
Isolate 3	0.26: 99.74	3.00: 94.45

## Discussion

The ability to produce high quality sequencing data from zoonotic parasites has direct and immediate implications for public and veterinary health. However, the variable amount of background host DNA in parasitic nematode samples can greatly reduce and sometimes entirely eclipse parasite signal [11] in whole genome sequencing. To provide an estimate of nematode signal in complex samples, we designed a robust and accurate qPCR assay (LupiQuant) with separate amplification and detection of parasite and host markers. The assay consists of cloned plasmid standards, each containing a single copy gene target from either the host or parasite. qPCR can then detect the host/parasite ratio that can be used to guide WGS efforts. Correlating WGS data with LupiQuant results showed a strong correlation (Fig 1), demonstrating the power of our approach.

One potential limitation to our approach is the presence of unexpected DNA in the sample (i.e., other pathogens co-infecting the host, contamination). For example, in one isolate from Flagstaff, Arizona, LupiQuant predicted the host-to-parasite DNA ratio as 53.25% host DNA and 46.75% parasite DNA. When the data were mapped against reference genomes, ~63% of the reads failed to align against dog or *O*. *lupi* references. When examining the ratio of mapped reads instead of total reads, the LupiQuant ratio estimate was correct. Blast results of the unaligned reads with the NCBI nt database identified what may be a fungal contaminant with less than 50% homology to any published organism. Additional testing on subsequent samples will determine if this contamination is isolated or widespread. The host-to-parasite ratio approach has been used previously for two tick-transmitted intracellular protozoal parasites, *Theileria annulata* and *Theileria parva*, both affecting cattle, but no report exists for filarial nematodes of the genus *Onchocerca* [16, 32]. Furthermore, this study is the first to use WGS data to validate the qPCR results. LupiQuant represents a critical method that allows researchers to selectively sequence *O*. *lupi*, conduct population structure studies to understand pathogen spread, develop diagnostics for accurate epidemiological surveillance, and potentially identify novel therapeutics to improve animal outcomes.

## Supporting information

S1 Table Accession information for polycystin-1 precursor (*pkd1*) gene sequences included for host locus primer design. (DOCX)

S2 Table Accession information for read data used for parasite locus primer design. (DOCX)
