# Supplementary material for: LupiQuant: A real-time PCR based assay for determining host-to-parasite DNA ratios of Onchocerca lupi and host Canis lupus from onchocercosis samples
Source: PLoS One. 2022 Nov 21;17(11):e0276916. doi: 10.1371/journal.pone.0276916 (PMC9678315; doi:10.1371/journal.pone.0276916)
Supplement: S1 Table — (DOCX) [file pone.0276916.s001.docx]

Table S1. Accession information for polycystin-1 precursor (*pkd1*) gene sequences included for host locus primer design.

| Gene Name | Accession Number |
| --- | --- |
| *Canis familiaris* polycystin-1 (*pkd1),* partial cds | AF483210.1 |
| *Canis lupus familiaris* breed Labrador retriever chromosome 06b | CP050622.1 |
| *Canis lupus familiaris* breed Labrador retriever chromosome 06a | CP050586.1 |
| *Canis lupus familiaris* polycystin 1, transient receptor potential channel interacting (PKD1), mRNA | NM_001006650.1 |
