# Supplementary material for: LupiQuant: A real-time PCR based assay for determining host-to-parasite DNA ratios of Onchocerca lupi and host Canis lupus from onchocercosis samples
Source: PLoS One. 2022 Nov 21;17(11):e0276916. doi: 10.1371/journal.pone.0276916 (PMC9678315; doi:10.1371/journal.pone.0276916)
Supplement: S2 Table — (DOCX) [file pone.0276916.s002.docx]

Table S2. Accession information for read data used for parasite locus primer design.

| Host | Species | Accession Number |
| --- | --- | --- |
| Cattle | *Onchocerca ochengi* | GCA_0009505151 |
| Cattle | *Onchocerca ochengi* | GCA_001077375 |
| Cattle | *Onchocerca ochengi* | GCA_900618345 |
| Deer | *Onchocerca flexuosa* | GCA_002249935 |
| Deer | *Onchocerca flexuosa* | GCA_900618345 |
| Dog | *Onchocerca lupi* | PRJNA802584 |
| Human | *Onchocerca volvulus* | GCA_000280695.1 |
